# The histone chaperone sNASP binds a conserved peptide motif within the globular core of histone H3 through its TPR repeats

**DOI:** 10.1093/nar/gkv1372

**Published:** 2015-12-15

**Authors:** Andrew Bowman, Lukas Lercher, Hari R. Singh, Daria Zinne, Gyula Timinszky, Teresa Carlomagno, Andreas G. Ladurner

**Affiliations:** 1 Department of Physiological Chemistry, Biomedical Center, Faculty of Medicine, Ludwig-Maximilians-Universität München, Großhaderner Strasse 9, 82152 Planegg-Martinsried, Germany; 2 Leibniz University Hannover, BMWZ—Institute of Organic Chemistry, Schneiderberg 38, 30167 Hannover, Germany; 3 Helmholtz Centre for Infection Research, Group of Structural Chemistry, Inhoffenstrasse 7, 38124 Braunschweig, Germany; 4 European Molecular Biology Laboratory, SCB Unit, Meyerhofstrasse 1, 69117 Heidelberg, Germany; 5 Center for Integrated Protein Science Munich (CIPSM), Department of Chemistry and Biochemistry, Ludwig-Maximilians-Universität München, Butenandt Strasse 5–13, 81377 Munich, Germany; 6 Munich Cluster for Systems Neurology (SyNergy), Ludwig-Maximilians-Universität München, Feodor Lynen Strasse 17, 81377 Munich, Germany

## Abstract

Eukaryotic chromatin is a complex yet dynamic structure, which is regulated in part by the assembly and disassembly of nucleosomes. Key to this process is a group of proteins termed histone chaperones that guide the thermodynamic assembly of nucleosomes by interacting with soluble histones. Here we investigate the interaction between the histone chaperone sNASP and its histone H3 substrate. We find that sNASP binds with nanomolar affinity to a conserved heptapeptide motif in the globular domain of H3, close to the C-terminus. Through functional analysis of sNASP homologues we identified point mutations in surface residues within the TPR domain of sNASP that disrupt H3 peptide interaction, but do not completely disrupt binding to full length H3 in cells, suggesting that sNASP interacts with H3 through additional contacts. Furthermore, chemical shift perturbations from ^1^H-^15^N HSQC experiments show that H3 peptide binding maps to the helical groove formed by the stacked TPR motifs of sNASP. Our findings reveal a new mode of interaction between a TPR repeat domain and an evolutionarily conserved peptide motif found in canonical H3 and in all histone H3 variants, including CenpA and have implications for the mechanism of histone chaperoning within the cell.

## INTRODUCTION

Eukaryotic genomes are packaged into chromatin. The composition and structure of chromatin fluctuates depending on the underlying genomic region, cell fate and environmental change, but nearly always contains nucleosomes as its basic repeating unit. The ability to properly assemble, disassemble and remodel nucleosomes is imperative for all cellular processes that require access to the DNA template, thus ensuring genomic stability.

Histone chaperones perform their vital role in genome maintenance by interacting with soluble histones, driving the accurate assembly and disassembly of nucleosomes (1,2). With respect to histones H3 and H4 in human cells, a growing number of chaperones, including ASF1, the HAT1 complex, NASP, the CAF1 complex, HIRA, DAXX, MCM2 and the FACT complex have been shown to interact with histones in their soluble, non-chromatin bound state (3–12). Interestingly, these histone chaperones represent a very diverse set of protein folds. Understanding how these different protein folds interact with their histone substrates is thus of great importance in understanding chromatin assembly and disassembly processes at the molecular level.

Some histone chaperones display isoform specific binding, such as the CAF1 complex for H3.1/.2 and the HIRA complex and DAXX for H3.3 (3,6–7), whilst others appear not to discriminate against histone H3 isoform, such as NASP, ASF1 and the HAT1 complex (3,13). More distantly diverged H3 isoforms, such as the centromeric variant CENPA, are also thought to have their own dedicated chaperones, such as Scm3/HJURP (14–16). For a number of pioneering cases, structural information regarding the interactions between histones H3 and H4 has been obtained, providing a mechanistic basis for their selective, or non-selective, binding and providing insights to how the histone chaperoning pathway may function at the molecular level (17–26).

One class of chaperone that has so far eluded structural analysis with its histone substrate is the tetratricopeptide repeat motif (TPR) containing family of histone chaperones represented by the human homologue NASP. In our study, we have addressed how sNASP recognizes its histone substrates and have identified a novel role for TPR repeat domains in histone binding. NASP was first identified as a protein expressed in testis (tNASP) (27) that interacted with the linker histone H1 (28). In dividing somatic cells a shorter splice isoform is prevalent (sNASP) (28), whereas both isoforms often occur in transformed cell lines (3). NASP was also shown to bind histones H3 and H4 *in vitro* and *in vivo* (3,7,11–12,29–32). Furthermore, N1/N2, a NASP homologue from *Xenopus laevis*, demonstrated strong binding to H3-H4 (33), as did the fission and budding yeast homologues Sim3 (344) and Hif1 (11,34–35), as well as the human protein (11,12). Moreover, NASP cooperates with ATP-dependent protein chaperones as well as Asf1, RbAp46 and the HAT1 complex in a histone folding and maturation program that effectively chaperones a reservoir of soluble H3-H4 for chromatin deposition during replication (11,32).

In this study we sought to identify how NASP engages its histone substrates. In particular, our attention fell on the conserved TPR repeat motifs present in NASP. Sequence analysis of NASP and its orthologues has revealed three canonical TPR repeat motifs (29,31,36–37) and a putative TPR motif interrupted by a large acidic region (38), a TPR capping helix C-terminal to TPR4 and an unstructured C-terminal region (36,37). tNASP contains a 339 residue insertion within the acidic domain, compared to sNASP, caused by splicing in of exon 6 (36,39). The functional relevance of the two isoforms in humans is currently unclear. However, eukaryotic homologues most closely resemble sNASP. These analyses have been supported by the recent crystal structure of the related protein Hif1p from yeast (40). Interaction studies between sNASP, H3, H4, H1 monomers and H3-H4 dimers using surface plasmon resonance suggested the most C-terminal TPR motif of sNASP is critical for the H3-H4 interaction (31), whereas pulldown experiments identified a region encompassing the N-terminal 80 residues of H3 as being sufficient to bind to NASP (32). Furthermore, a V71M mutation in the testicular H3 variant H3T was proposed to inhibit the chaperoning activities of sNASP *in vitro* (41). The molecular identity of the H3/H4 ligand for the TPR repeats and whether sNASP makes multivalent interactions with H3-H4 thus remain unclear.

We thus decided to dissect both the regions of NASP and of the histones H3-H4 that are key to the known interactions between this chaperone and its histone substrates using biophysical, live cell imaging and structural biology techniques. We demonstrate that sNASP interacts with an evolutionarily conserved heptapeptide epitope located within the globular core of the histone fold of H3. The core motif is comprised of the residues L126, A127, I130, R131 and G132, which are found in the C-terminal helix of folded H3, and is largely conserved throughout evolution and across histone variants, including the diverged centromeric variants of H3 (CenpA and its orthologues). Binding occurs through an archetypal TPR—peptide mode of interaction, with a small region juxtaposed to the C-terminal end of the TPR domain also being necessary for binding. Overall, our findings identify a novel TPR-peptide interaction with histone H3 and a new perspective on how histones are engaged by components of the histone chaperoning network.

## MATERIALS AND METHODS

### Protein expression and purification

sNASP was expressed in bacteria as an N-terminal (His)_6_ fusion construct and was purified over Nickel-NTA beads (GE Healthcare) in 300 mM sodium chloride, 50 mM Tris–HCl pH 8.0, 0.1% NP40, 20 mM imidazole and Complete protease inhibitor cocktail (Roche). Protein was eluted in the same buffer with 250 mM imidazole, but without protease inhibitors. The (His)_6_ tag was cleaved overnight by TEV protease, whilst concomitantly dialysing to 100 mM sodium chloride, 20 mM Tris–HCl pH 8.0, 1 mM EDTA and 5 mM dithiothreitol. Cleaved protein was further purified by ion-exchange using a ResourceQ column (GE Healthcare) before gel filtration chromatography in 200 mM sodium chloride, 20 mM Hepes-KOH pH 7.6 and 1 mM EDTA using a 26/60 Superdex 200 column (GE Healthcare). The purified protein was concentrated and stored in aliquots at −80°C. Homologues, truncations and point mutants of sNASP were expressed and purified using the same method. MBP-H3 peptide fusions were expressed in bacteria and used as whole cell extracts (epitope mapping) or purified over a Dextrin Sepharose column (GE Healthcare) in one-step purification in 0.5 M sodium chloride, 20 mM Hepes-KOH pH 7.6, 1 mM EDTA and 5 mM dithiothreitol. Concentrated protein was dialysed against 200 mM sodium chloride, 20 mM Hepes-KOH, 1 mM EDTA and 5 mM dithiothreitol and stored in aliquots at −80°C.

### Isotope labelled sNASP production for NMR anlaysis

^15^N, ^13^C, ^2^H labelled protein for nuclear magnetic resonance (NMR) analysis was essentially performed as previously described (42). Briefly, three colonies transformed with the sNASP (41–330, Δ101–159) expression cassette were grown in 5 ml M9 minimal medium supplemented with ^15^NH_4_Cl, ^13^C_6_-glucose and 50% D_2_O for 9 h. A total of 2.5 ml of cells were then collected by centrifugation and grown overnight at 37°C in the same medium, but supplemented with ^15^NH_4_Cl, ^13^C_6_-glucose and 75% D_2_O. The next day 200 ml was removed and used to inoculate 5 ml of fresh medium, but supplemented with ^15^NH_4_Cl, ^13^C_6_-glucose and 99.9% D_2_O. After 9 h of growth at 37°C, 0.5 ml of culture was taken to inoculate 90 ml of fresh media containing ^15^NH_4_Cl, ^13^C_6_-glucose and 99.9% D_2_O and grown overnight at 37°C. This culture was then used to inoculate a 2 l expression culture supplemented with ^15^NH_4_Cl, ^13^C_6_-glucose and 99.9% D_2_O. When the culture reached an optical density at 600 nm of 0.8, protein expression was induced using 0.1 mM IPTG. Protein was expressed for 20 h at 20°C. For singly labelled ^15^N protein, minimal media was supplemented only with ^15^NH_4_Cl. Three colonies were grown in 20 ml LB medium over 3 h at 37°C before collection by centrifugation and resuspension in 100 ml of M9 minimal medium supplemented with ^15^NH_4_Cl_._ The next day this culture was used to inoculate 1 l of the same medium and grown at 37°C. At an optical density at 600 nm of 0.8, protein expression was induced by at 0.1 mM IPTG. Expression was carried out at 20°C for 18 h. Labelled protein was purified in the same way as unlabelled sNASP.

### Far-western blotting

sNASP carrying an N-terminal AviTag was biotinylated using recombinant BirA from *Escherichia coli*. Whole cell extracts were prepared from U2OS cells, separated by sodium dodecyl sulphate-polyacrylamide gel electrophoresis (SDS-PAGE) and blotted onto nitrocellulose. Membranes were probed with 1 μM biotinylated sNASP in TBST supplemented with 3% dried milk or with an H3 antibody (abcam—ab1791). Detection of bound sNASP was carried out using streptavidin-HRP conjugate (Invitrogen). After imaging, membranes were reprobed with an H4 antibody (abcam—ab10158).

### Analytical gel filtration

Analytical gel filtration was carried out using a Superdex S200 10/300 Gl column (GE Healthcare) in 20 mM Hepes-KOH pH 7.5 and 200 mM sodium chloride, unless otherwise stated. 0.5 ml fractions were collected encompassing the void (7 ml) and bed (21 ml) volumes of the column. Fractions were separated by SDS-PAGE and stained with Coomassie Brilliant Blue (SERVA).

### Isothermal titration calorimetry

Purified sNASP and MBP H3 116–135 were dialysed extensively against 20 mM Hepes-KOH, pH 7.5 and 200 mM sodium chloride. Isothermal titration calorimetry (ITC) was carried out using a MicroCal iTC200 (GE Healthcare). sNASP was used as titrant at 180, 150 and 100 μM, and injected into the cell containing MBP H3 116–135 at 30, 15 and 10 μM respectively, in three independent experiments. Isotherms were analysed by the software NITPIC and SEDPHAT (43) and plotted using the software GUSSI.

### Epitope mapping of the C-terminus of H3

Mutations were made in the MBP H3 116–135 construct using a mixture of site-direct mutagenesis and oligo ligation. Whole cell extracts were prepared from bacteria and used as inputs for the pulldown experiments with (His)_6_-sNASP pre-bound to Nickel-NTA beads (GE Healthcare). Pulldown of MBP H3 116–135 by triple point mutants of sNASP was carried out in the same way, except higher stringency washing was carried out in 50 mM potassium hydrogen phosphate pH 7.5, 300 mM sodium chloride, 0.1% NP40 and 20% imidazole.

### Fluorescence-2-hybrid assay

For all the F2H assays, we used human U2OS cells harbouring the stably integrated lacO (256×) array described earlier (44), except for mitochondrial F2H, which was carried out on U2OS cells without an integrated array. F2H assays were essentially performed as previously described in (45,46) and (44). Image analysis was manually performed with ImageJ image analysis software. Fluorescence intensity at the LacI array was calculated as a percentage increase over the nucleoplasm. Assignment of the LacO array and nucleoplasm were done manually. Twenty cells were counted for statistical analysis so as not to inflate the calculated *P*-value. Only cells in which a clearly discernable array was present were counted. For the mitochondrial F2H, the first 70 residues of the outer mitochondrial membrane protein TOM70 were cloned onto the N-terminus of mCherry. Full length H3 or H3 residues 116–135 was then cloned onto the C-terminus of mCherry to create a TOM70-mCherry-Histone triple fusion. Deleting residues 378–383 using standard site-directed mutagenesis techniques disrupted the nuclear localization sequence (NLS) of mEGFP-sNASP, resulting in its cytoplasmic expression. Mitochondrial targeted histones and cytoplasmic sNASP constructs were cotransfected into U2OS cells and colocalization to the mitochondrial network was observed by fluorescence microscopy.

### NMR analysis of sNASP

NMR experiments were performed on Bruker Avance III 800 MHz and 600MHz spectrometers, both equipped with HCN triple-resonance cryogenic probes. NMR data were recorded at a temperature of 298 K with a sample buffer of 20 mM NaPO_4_ pH 7.0, 100 mM NaCl and 5 mM β-mercaptoethanol. Assignment experiments (TROSY-enhanced HNCA, HN(CO)CA, HN(CA)CB & HN(COCA)CB) were performed on a 70% deuterated, uniformly ^13^C, ^15^N-labelled sNASP (41–330, Δ101–159) at a concentration of 0.72 mM with 1.2 equivalents H3 115–135 peptide (Anaspec, 61704). About 95% of the backbone HN resonances were assigned for the peptide-bound state. Due to slow exchange between the free and bound states, it was not possible to directly transfer assignments from the peptide–sNASP complex to apo-sNASP from the ^15^N-TROSY-HSQC spectra alone. An additional TROSY-enhanced HNCA spectrum for free sNASP (0.36 mM, in identical buffer) allowed unambiguous transfer of assignments for 99% of the previously assigned residues. This allowed unambiguous transfer of assignments for 99% of the previously assigned residues. Data were processed in Topspin (Bruker) and analysed using CCPNMR analysis (47). CSPs were calculated by CCPNMR analysis and visualized using gnuplot (http://www.gnuplot.info). Structures are visualized using PyMol (48). Peptide titrations were performed with protonated ^15^N-labelled sNASP (41–330, Δ101–159) in PBS using the same H3 peptide (H3 115–135: KRVTIMPKDIQLARRIRGERA) (Anaspec) used for assignment.

## RESULTS

### sNASP interacts with the C-terminal α3 helix of histone H3

**Figure 1.**
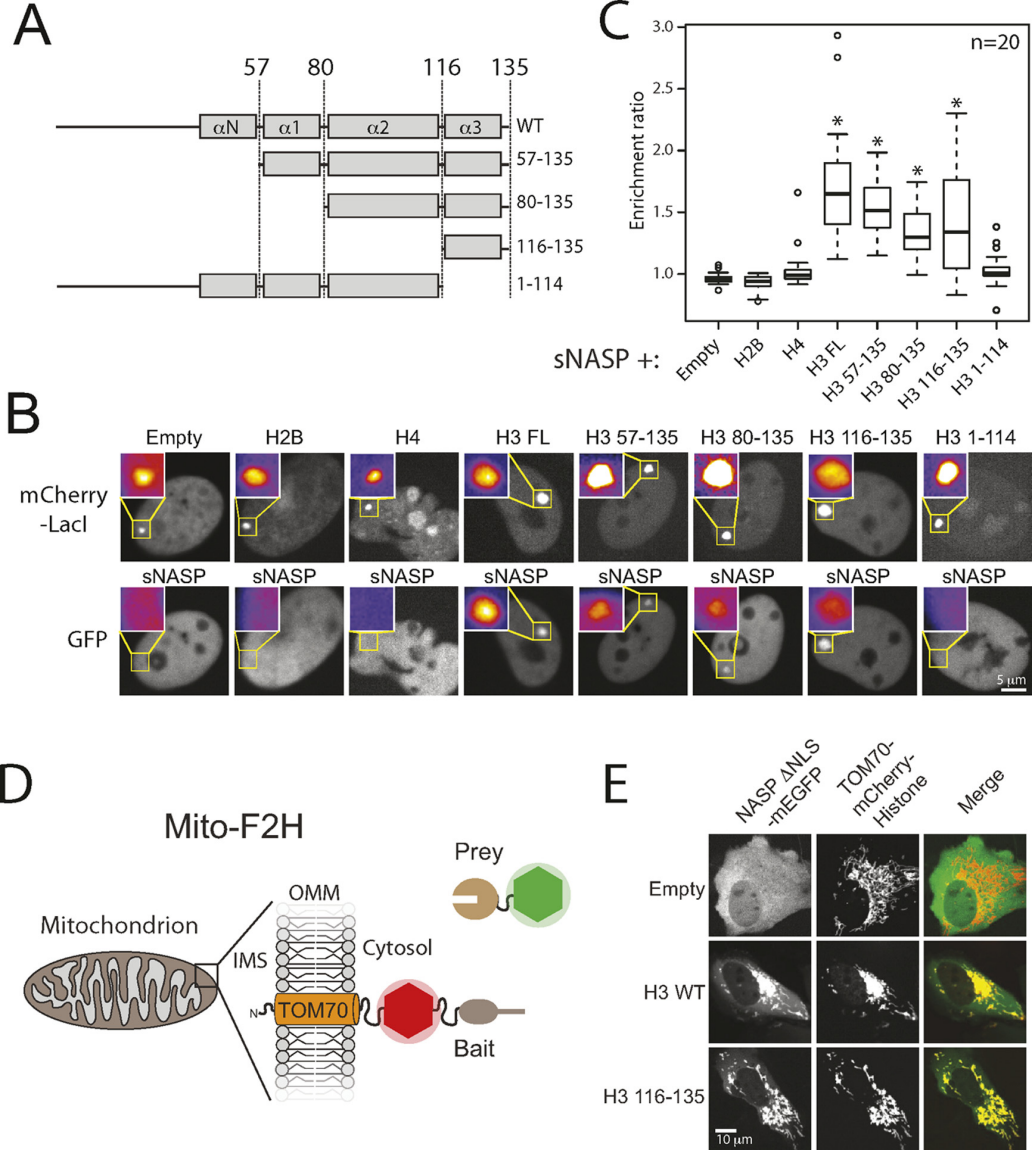
sNASP interacts with a conserved C-terminal region in histone H3. (**A**) The H3 truncation constructs used for sNASP interactions analysis using the F2H assay. Residue numbers of each fragment are displayed to the right. The regions shown were expressed as a C-terminal fusion to mCherry-LacI. (**B**) F2H analysis of the interaction between EGFP-sNASP and mCherry-LacI-Histone constructs. Inset represents magnified regions of the LacO array, smoothed and coloured to aid visualization. The images shown are representative samples of those quantified in (B). (**C**) Quantitation of experiments shown in (B). Boxes represent the lower quartile, median and upper quartile. Whiskers represent 1.5× the interquartile range. Outliers are indicated by circles. Asterisks indicate a significant difference in enrichment compared to the empty vector control (*P*-value < 0.007, Wilcoxon rank sum test). (**D**) Diagramatic representation of the mitochondrial F2H system. Fusion of the first α-helix of human TOM70 to a mCherry-bait protein allows the mitochondrial network to be used as an F2H platform. OMM, *Outer Mitochondrial Membrane*; IMS, *Inner Membrane Space*. (**E**) Cytoplasmic sNASP interacts with both full length H3 and H3 residues 116–135 when fused to the outer surface of mitochondria, but does not recruit to the TOM70-α1-mCherry alone.

TPR proteins often play scaffolding roles, mediating protein–protein interactions through binding to short peptide motifs (49,50). As the histone chaperone sNASP contains multiple TPR motifs, we wondered whether it interacts with its histone substrate through a canonical TPR-peptide interaction. In an initial screen we used biotinylated sNASP to probe whole extracts from U2OS cells in a far western blot experiment, detecting a specific band corresponding to endogenous histone H3, but not to H4 (Supplementary Figure S1). It is also worth noting that we do not see a prominent higher molecular weight band corresponding to the size of H1. However, this may be due to the non-native nature of displayed proteins in a far western blot. To further define the region of H3 recognized by sNASP we employed the F2H assay to screen different histone fragments for sNASP binding in live cells (Figure 1A–C). The F2H assay is a powerful approach for screening binary interactions between two proteins of interest through induced colocalization to an integrated LacO array (45,46). As the F2H assay takes place in the heterogeneous environment of the cell nucleus, binding likely has to be highly specific in order for an interaction to be observed. This assay revealed interaction between full-length H3 and sNASP, but not with H4 or H2A (used as controls), with fragmentation of H3 identifying a short region encompassing the last 20 residues of H3 as being sufficient for binding. Furthermore, sNASP did not interact with a fragment representing all but the last 21 residues of H3 (H3 1–114), demonstrating that the sNASP-H3 peptide interaction is specific to a region encompassing the C-terminal helix of H3.

Histones are DNA packaging proteins. Using genomic loci as a tethering platform for the analysis of histone–chaperone interactions could thus be confounded by their local genomic environment. To focus solely on the binary interaction between histones and chaperones, independent of the environmental context, we thus developed a F2H assay outside of the nucleus and employed a novel mitochondrial scaffold for interaction analysis in the cytoplasm. An N-terminal fusion of the first α-helix of TOM70 has previously been shown to tether a fluorescent protein to the outer surface of mitochondria (51). We reasoned that this approach could be used to assess protein-protein interactions in the cytoplasm by tethering a bait protein to the outer mitochondrial surface and testing for colocalization of a soluble tagged prey protein (Figure 1D). To test this, we tethered mCherry alone, mCherry fused to H3 or mCherry fused to the H3 116–135 peptide to the α1 helix of TOM70 and looked for recruitment of soluble GFP-tagged sNASP. Human sNASP bears a strong NLS (52) (Figure 1B). Therefore to observe interaction in the cytoplasm we first deleted the NLS of sNASP (residues 378–383), which resulted in its cytoplasmic expression. Cytoplasmic sNASP showed a strong recruitment to both full length α1-TOM70-mCherry-H3 and α1-TOM70-mCherry-H3 116–135, but not to α1-TOM70-mCherry alone (Figure 1E). This finding suggests that sNASP interacts with its H3 C-terminal peptide independent of the nuclear environment, implying that it binds directly to the H3 peptide. Our findings reveal that sNASP interacts with a short peptide region at the terminal end of the H3 protein.

### sNASP binds the H3 C-terminal peptide *in vitro* with nanomolar affinity

**Figure 2.**
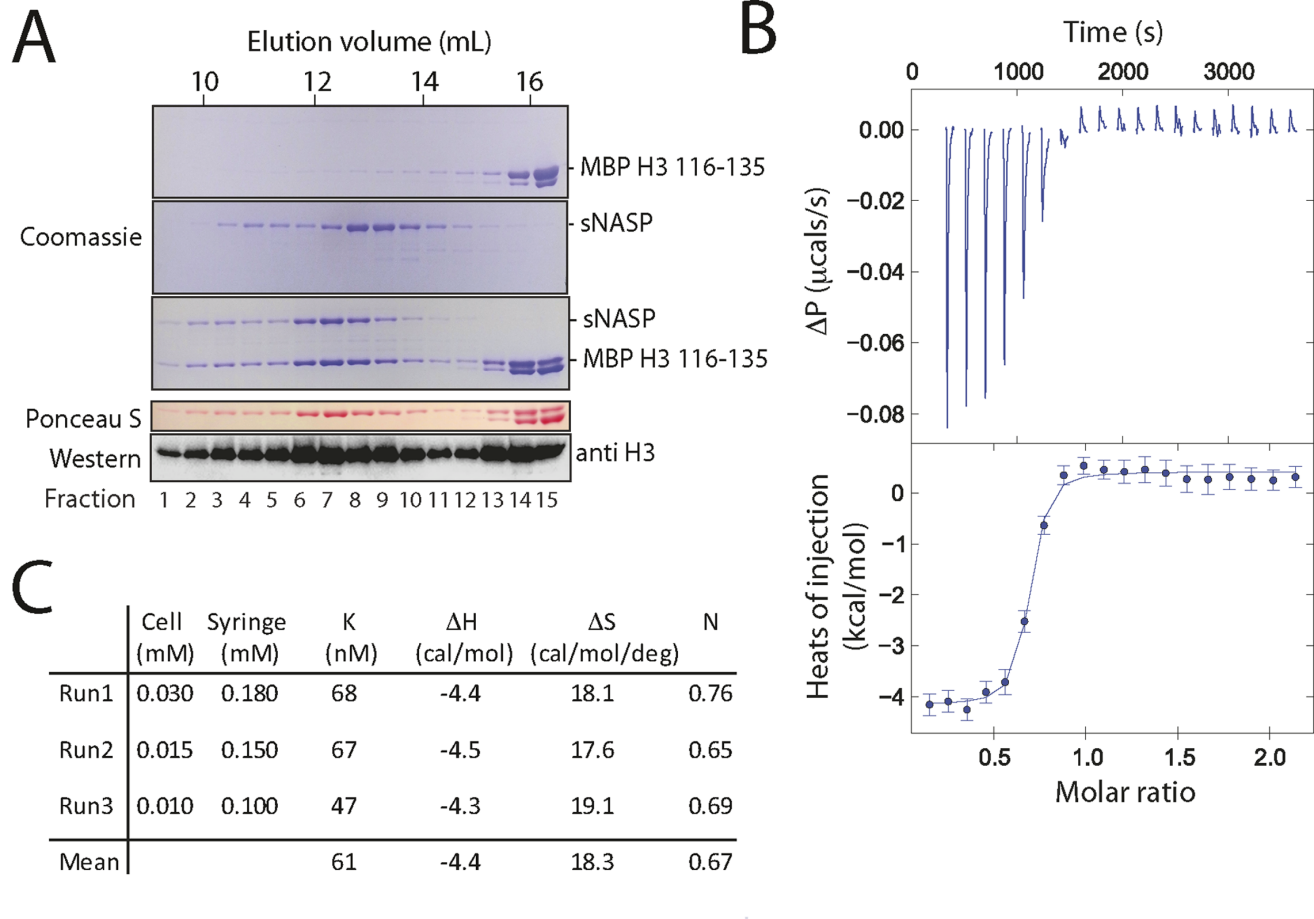
sNASP interacts with the C-terminal region of H3 with high affinity. (**A**) Complex formation between sNASP and the 20 C-terminal residues of H3 fused to maltose binding protein (MBP H3 116–135) demonstrated by size exclusion chromatography. (**B**) Quantification of the interaction between sNASP and the 20 C-terminal residues of H3 by isothermal titration calorimetry (ITC). An example of a typical thermogram (top) and isotherm (bottom) is shown. Integration of injection peaks was carried out using the program NITPIC. The data were fitted using SEDPHAT and plotted using the program GUSSI. (**C**) Values from three independent ITC experiments. sNASP was added to the syringe and injected into a sample cell containing MBP H3 116–135.

We next sought to characterize the sNASP-H3 peptide interaction *in vitro*. We found that fusion of the C-terminal 20 residues of H3 to MBP (MBP H3 116–135) was soluble when expressed recombinantly in bacteria, and used this construct to probe the interaction with sNASP in more detail. Gel filtration analysis of recombinant sNASP and MBP H3 116–135 revealed that they form a stable complex under physiologically relevant buffer conditions, and further confirmed that the interaction is direct and not bridged by other factors (Figure 2A). To quantify the stoichiometry and strength of interaction between sNASP and H3 116–135, ITC was carried out using the MBP H3 116–135 fusion protein. Titrating sNASP into MBP H3 116–135 gave a dissociation constant of 47–68 nM (*n* = 3 with varying cell and titrant concentrations; Figure 2B and C), fitting a one-site binding model with close to 1:1 stoichiometry (partial degradation observed during protein purification likely caused the observed molar binding ratios to be underestimated, as is often the case). Such high affinity is surprising as peptide binding domains usually bind with dissociation constants in the low to mid micromolar range. Whilst a mid nanomolar dissociation constant is on the upper confidence limit for accurate determination by ITC, it is clear that *in vitro* sNASP interacts with its H3 peptide ligand with unusually high affinity.

### Epitope mapping of sNASP's binding site reveals a conserved five-residue motif in H3

**Figure 3.**
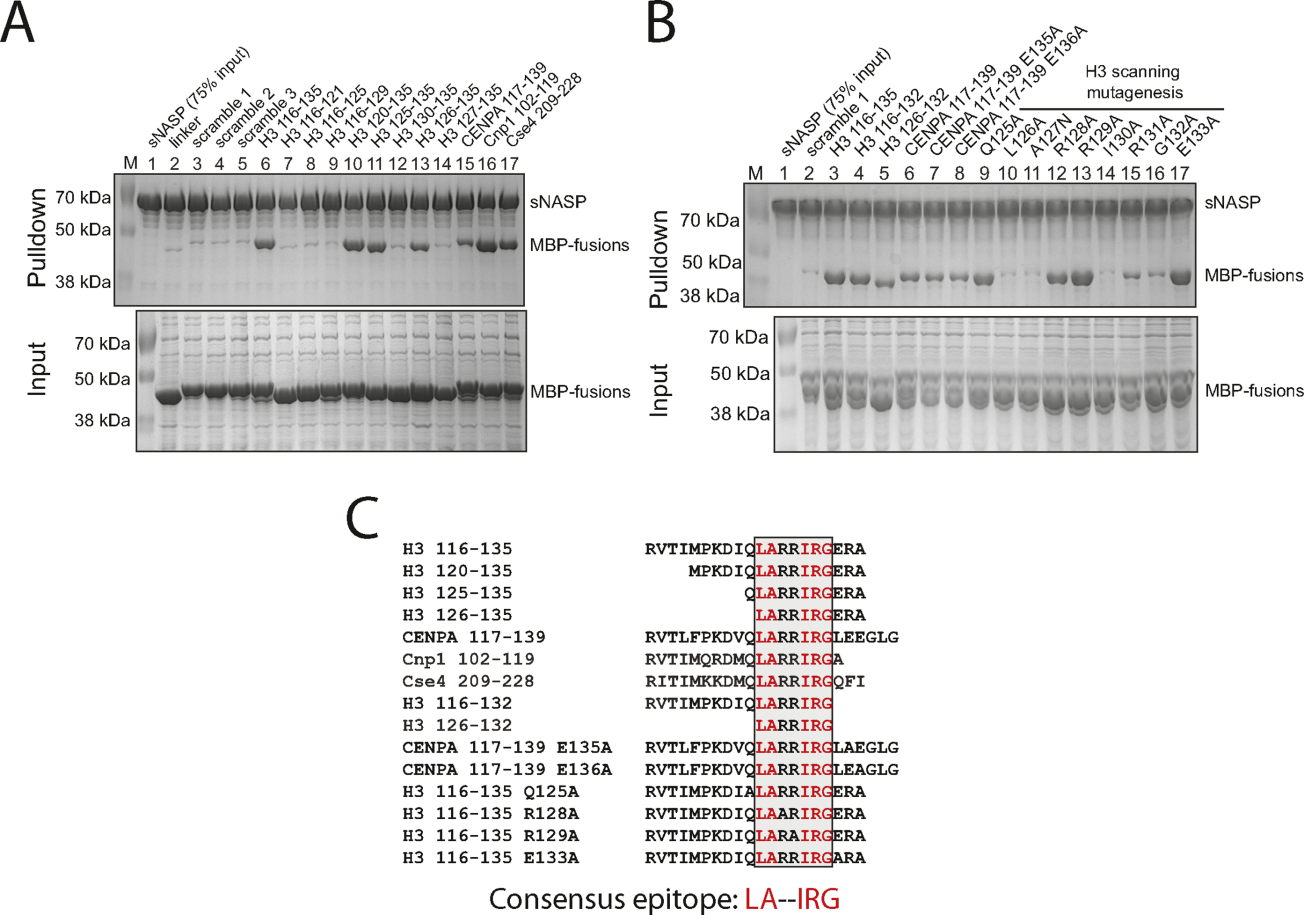
Mapping the H3 epitope of sNASP reveals a conserved heptapeptide motif as being necessary and sufficient for interaction. (**A**) Pulldown of H3 truncations and H3 variants fused to the C-terminus of MBP by (His)_6_-tagged sNASP. The positions of sNASP and MBP-fusion are marked. Components of the pulldowns were separated by SDS-PAGE and stained with Coomassie. (**B**) Pulldown of further truncations of H3 and CENPA and scanning mutatgenesis of the region 125–133 of H3. (**C**) Alignment of all peptides that displayed interaction with sNASP. Residues common to all are highlighted in red, with the consensus epitope shown expanded underneath.

To further define the interaction site within the C-terminus of H3, we expressed a variety of truncations fused to MBP with a 20 amino acid linker and determined the ability of (His)_6_-sNASP to pull down these truncations from whole cell bacterial extracts. Whilst being unable to pull down scrambled or linker peptide fusions, MBP-H3 116–135 (wild-type) was pulled down to near stoichiometric amounts (Figure 3A). Fragments corresponding to residues (120–135 and 125–135 were also pulled down efficiently, while fragments 130–135, 116–120, 116–125 or 116–130 were not, showing that the sNASP binding epitope must reside within the final 10 amino acids of H3. Further truncation analysis revealed that elimination of Q125 has limited effects on binding, but that removal of L126 results in complete loss of interaction (Figure 3A, lanes 13 and 14). As the C-terminal region of H3 is highly conserved between species and is also highly conserved in histone H3 variants, we tested the ability of sNASP to pulldown homologous regions of the CENPA variant of H3 from human (CENPA), budding yeast (Cse4) and fission yeast (Cnp1). Remarkably, sNASP is able to pull down all three homologues (Figure 3A, lanes 15–17), identifying that sNASP and its homologues may be important in chaperoning all isoforms and variants of H3, as has been suggested for the fission yeast homologue Sim3 (38).

The C-termini of proteins can be important in binding interactions, especially in TPR motif-mediated interactions (53,54). As the sNASP binding epitope in H3 lies very close to the C-terminus we wanted to address whether the C-terminal carboxylate is important for interaction. Variability in the length of this region in the CENPA homologues argues against the importance of the C-terminus, although the only homologue that extends past the canonical H3 sequence is human CENPA, which contains two glutamic acid residues that could possibly act as C-terminal carboxyl mimics. To test for C-terminal positioning effects, a protein fusion corresponding to H3 residues 116–132, and 126–132, were tested alongside the E135A and E136A mutants of CENPA. CENPA point mutants pull down to a similar extent to that of CENPA 119–137, suggesting that these residues do not contribute significantly to the sNASP interaction (Figure 3B, lanes 6–8). H3 116–132 pulls down slightly less efficiently than H3 116–135, and 126–132 even less so (Figure 3B, lanes 3–5), but still significantly above background. This indicates that residues flanking H3 126–133 may contribute to sNASP binding, but are not essential. Furthermore, analysis of sNASP's protein sequence shows that it does not contain a motif that is typical for TPR proteins that bind to C-terminal carboxylates (54).

Finally, scanning mutagenesis was carried out on the remaining heptapeptide LARRIRG consensus sequence. As expected, mutation of the two residues flanking the heptapeptide motif, Q125A and E133A, had no observable effect on binding (Figure 3B). Neither did mutation of the two central arginines, R128A and R129A. In contrast, mutations L126A, A127N, I130A and G132A resulted in complete loss of binding to sNASP, with R131A showing much reduced binding ability (Figure 3B). Aligning all peptides that display interaction with sNASP resulted in a consensus sequence in which the five amino acids essential for sNASP interaction are identical across all variants and homologues tested, and are highly conserved throughout eukaryotic evolution (Figure 3C).

### H3 C-terminal peptide binding is widely conserved amongst sNASP homologues

**Figure 4.**
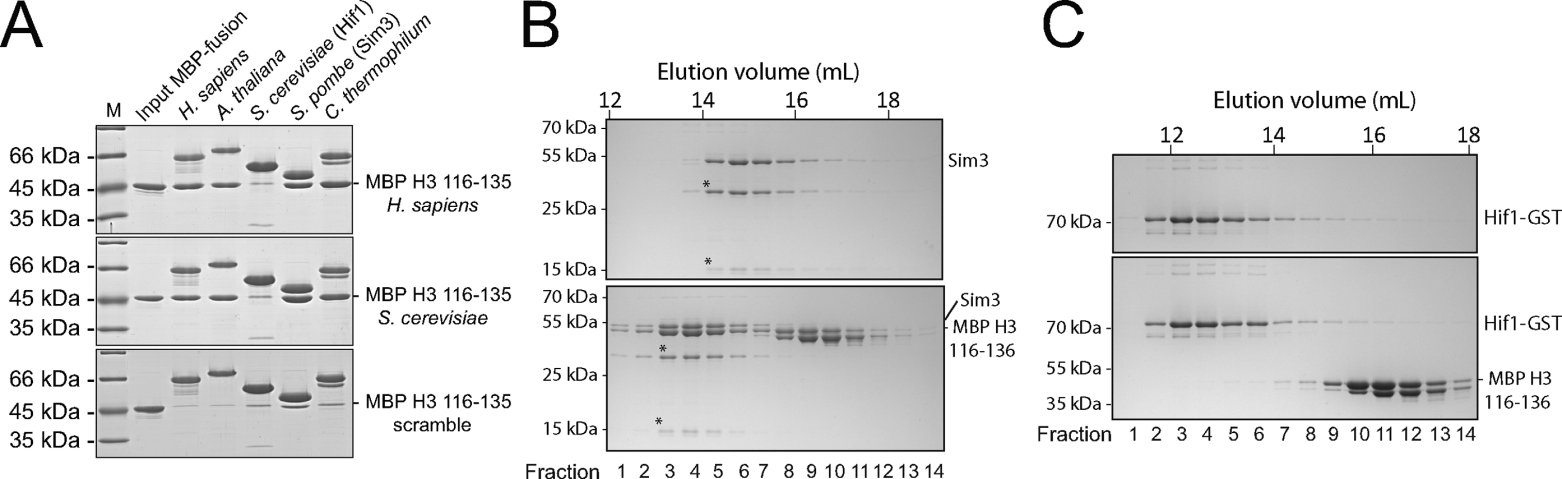
H3 peptide binding by the histone chaperone NASP is widely conserved from plants to humans, but not by the budding yeast homologue Hif1. (**A**) The ability of sNASP homologues to pull down MBP H3–116–135. Both the *Homo sapiens* (top) and *Saccharomyces cerevisiae* (middle) H3 sequences were tested, along with a scrambled peptide as a control (bottom). (**B**) Interaction between Sim3 and MBP H3 116–135 analysed by size exclusion chromatography. Asterisks indicate fragments of Sim3, most likely resulting from non-specific cleavage within its acidic domain. (**C**) Interaction between GST-tagged Hif1 and MBP H3 116–135 analysed by size exclusion chromatography. GST-tagged Hif1 was used to resolve the two eluting species, as untagged or six-histidine tagged Hif1 eluted very close to the MBP H3 116–135 fusion.

As sNASP interacted with a broad range of H3 isoforms present in both humans and yeasts, we wondered how far in evolution the sNASP-H3 peptide is conserved. To answer this, homologues of sNASP from *Arabidopsis thaliana, Schizosaccharomyces pombe, Chaetomium thermophilum* and *Saccharomyces cerevisiae* were identified by BLAST analysis, cloned and purified as (His)_6_-tagged recombinant proteins in bacteria. These were then tested for their ability to pull down MBP H3 116–135. Whilst homologues from *A. thaliana, S. pombe* (Sim3) *and C. thermophilum* pulled down stoichiometric amounts of the MBP H3 116–135 peptide fusion, no pull down was observed for the *S. cerevisiae* homologue (Hif1) (Figure 4A, top panel). This was not due to minor differences in flanking amino acids of *S. cerevisiae* H3 as compared to the human H3 sequence, as Hif1 also failed to pull down the corresponding region of the *S. cerevisiae* H3 homologue (Figure 4A, middle panel), whereas binding of the other homologues was not perturbed. None of the homologues pulled down a scrambled H3 peptide fusion (Figure 4A, bottom panel). To further validate the interactions, Sim3 and Hif1-GST were mixed with MBP H3 116–135 and their ability to form a stable complex was assessed by gel filtration chromatography (Hif1 was GST tagged to shift its elution profile so that it could be resolved from MBP H3 116–135). Whilst Sim3 coeluted with MBP H3 116–135, as expected, Hif1-GST and MBP H3 116–135 eluted in separate fractions (Figure 4B and C, respectively). These results indicate that Hif1 may be functionally distinct from other sNASP homologs. Interestingly, Hif1 has been shown to make a direct interaction with the Hat1–Hat2 complex (11,34), whereas in humans interaction of sNASP with HAT1-RbAp46 (the human Hat1-Hat2 homologues) was shown to be bridged by histones (11). One possible explanation for the loss of binding function we see from recombinant Hif1 could be explained by a gain of function relating to Hif1's interaction with the Hat1–Hat2 complex. In any case, our findings suggest that interaction between the C-terminal region of H3 and the NASP family of histone chaperones appeared early on in the evolution of eukaryotes and is a widespread ligand-binding feature of this conserved histone chaperone.

### Mapping the H3 binding interface of sNASP by NMR spectroscopy

In addition to characterising the interaction biochemically, we set out to gain structural insights into how sNASP interacts with the C-terminal H3 peptide. As to date we have been unable to obtain crystals of the sNASP-H3 peptide complex, we used NMR spectroscopy to monitor the binding of H3 to sNASP. Previous sequence analysis of sNASP and its homologues has shown they contain four TPR motifs, a large acidic domain that interrupts TPR2 and a region of α-helical structure predicted to form a ‘capping’ helix typical of TPR repeat proteins (see Supplementary Figure S2B for a domain diagram of sNASP) (38,36). Additionally, although we couldn't detect any binding to the H3 C-terminal peptide, as shown earlier, these predictions were largely confirmed by the recent crystal structure of the budding yeast homologue Hif1 (40).

To aid in NMR analysis we investigated whether the domain of sNASP responsible for binding the H3 peptide could be further defined. To test this, a number of (His)_6_-tagged truncated proteins were created and their ability to pull down MBP H3 116–135 was assessed (Supplementary Figure S2). Interestingly, whilst a region encapsulating TPR 2, 3, 4 and the capping region were necessary for H3 peptide binding, the acidic domain was not. Interestingly, a contiguous TPR (cTPR) mutant is able to pull down MBP H3 116–135 similar to wild type (Supplementary Figure S2A and B). Truncating sNASP close to TPR4 at residue 300 resulted in the loss of interaction, but truncation at residue 330 retained the interaction, suggesting that the region C-terminal to the TPR domain, covering residues 300–330, is also important for binding (Supplementary Figure S2A and B). Assimilating these findings, we derived a construct spanning residues 41–330 (including all of the TPR motifs and the predicted coiled-coil domain), with an internal deletion of residues 101–159 (the majority of the acidic domain), that was stable and monodisperse at high concentrations and retained its interaction with the H3 peptide (Supplementary Figure S2B and C).

The ^15^N-^1^H resonances of this construct showed excellent dispersion and were assigned using standard NMR methods (see ‘Materials and Methods’ section). To confirm that the structure of sNASP is similar to that of other TPR domains, we compared the backbone chemical shifts (Cα/Cβ) of sNASP41–330 (Δ101–159) with the secondary structure elements predicted from a homology model of the TPR domain of sNASP. The model was generated using the crystal structure of the budding yeast homologue Hif1 as a template (Supplemental Figure S3) in the model, the TPR motifs adopt the positions predicted from previous bioinformatics analysis of TPR repeat proteins (49,54–55). In contrast, the capping region C-terminal to the TPR motifs, had little homology to the corresponding region of the Hif1 crystal structure, and was modelled with less confidence as a ‘capping’ α-helix and a 14 amino acids insertion (Supplemental Figure S3). Analysis of Cα/Cβ secondary chemical shifts (56) indicated that the last TPR4 α-helix and the capping region are both extended by 5–6 amino acids: helicity in this regions was incorporated in the model. Overall, the calculated chemical shift index showed an excellent match with the secondary structure elements of the sNASP model.

**Figure 5.**
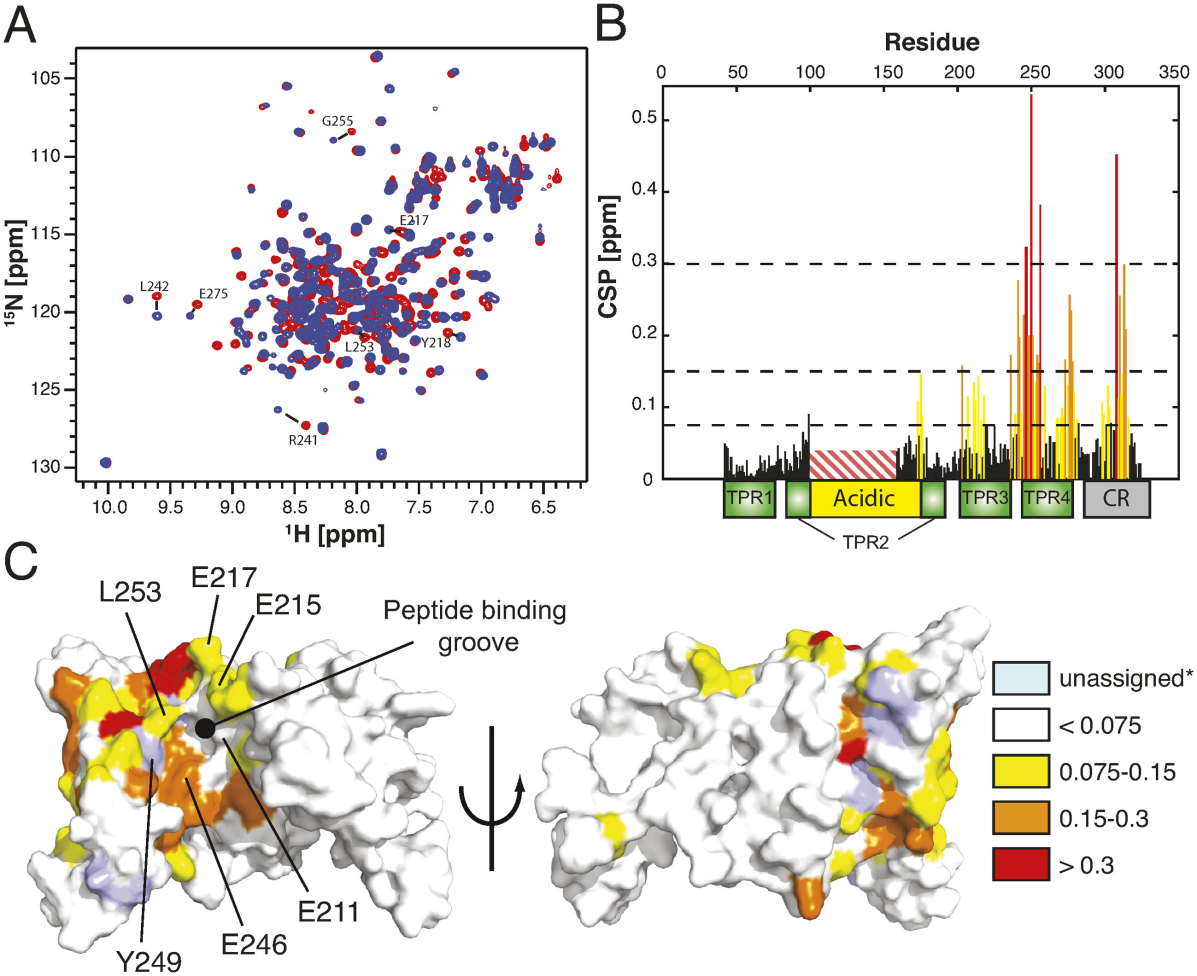
The H3 peptide binds in the central channel formed by sNASP's TPR and capping region. (**A**) Overlay of ^1^H-^15^N HSQC spectra of the apo (red) and H3 peptide (115–135) bound sNASP (41–330, Δ101–159) (blue). Some well-separated peaks located on the proposed interaction surface are marked. (**B**) Weighted chemical shift perturbations are plotted against residue position in sNASP. The domain architecture is given below the plot and the deleted acidic region in sNASP (41–330, Δ101–159) is indicated. Colouring was done using the same key as in panel C except that black is used for CSP below 0.75 ppm. (**C**) Observed CSP mapped on the structural model of sNASP. CSP values are in ppm. The concave surface of the central channel formed by the stacked TPR motifs and the capping region (left image) shows enrichment in CSPs compared to the convex outer surface (right image). The positions of point mutants from Figure 6 that disrupted binding to the H3 peptide are indicated. *Residues that could not be assigned in the peptide bound state or that could not be unambigiously assigned in apo-sNASP are marked as unassigned.

Titration of sNASP with an H3 peptide spanning residues 115–135, revealed tight binding in the slow exchange regime, in agreement with the nanomolar dissociation constant previously determined by ITC for a larger sNASP construct (Figure 2B). As predicted from the biochemical analysis, the largest chemical shifts perturbations (CSP) map to the region spanning TPR3, TPR4 and the predicted capping region C-terminal to TPR4 (Figure 5B). Mapping the observed CSPs onto the structural model revealed that the largest CSPs cluster in the concave central channel of the TPR domain and in the capping α-helix, whereas the convex outer surface is much less affected by peptide binding (Figure 5C). In agreement with the CSPs, the electrostatic potential of the central channel shows a negatively charged surface largely overlapping with the interaction surface (Supplemental Figure S3B and C): the charges of the channel are nicely complementary to the basic side-chains within the H3 peptide ligand.

We have previously observed that while the N-330 sNASP truncation binds the H3 C-terminal peptide with affinity similar to the full length, the N-300 truncation is incapable of binding H3 (Supplementary Figure S2A). In our analysis we see a large CSPs for residue 302–315, confirming the importance of these residues for the sNASP–H3 interaction. Taken together, our NMR analysis confirms that the central channel of the TPR domain and the juxtaposed capping region form the interaction surface for the H3 C-terminal peptide.

### H3 peptide binding maps to the TPR domain of sNASP

**Figure 6.**
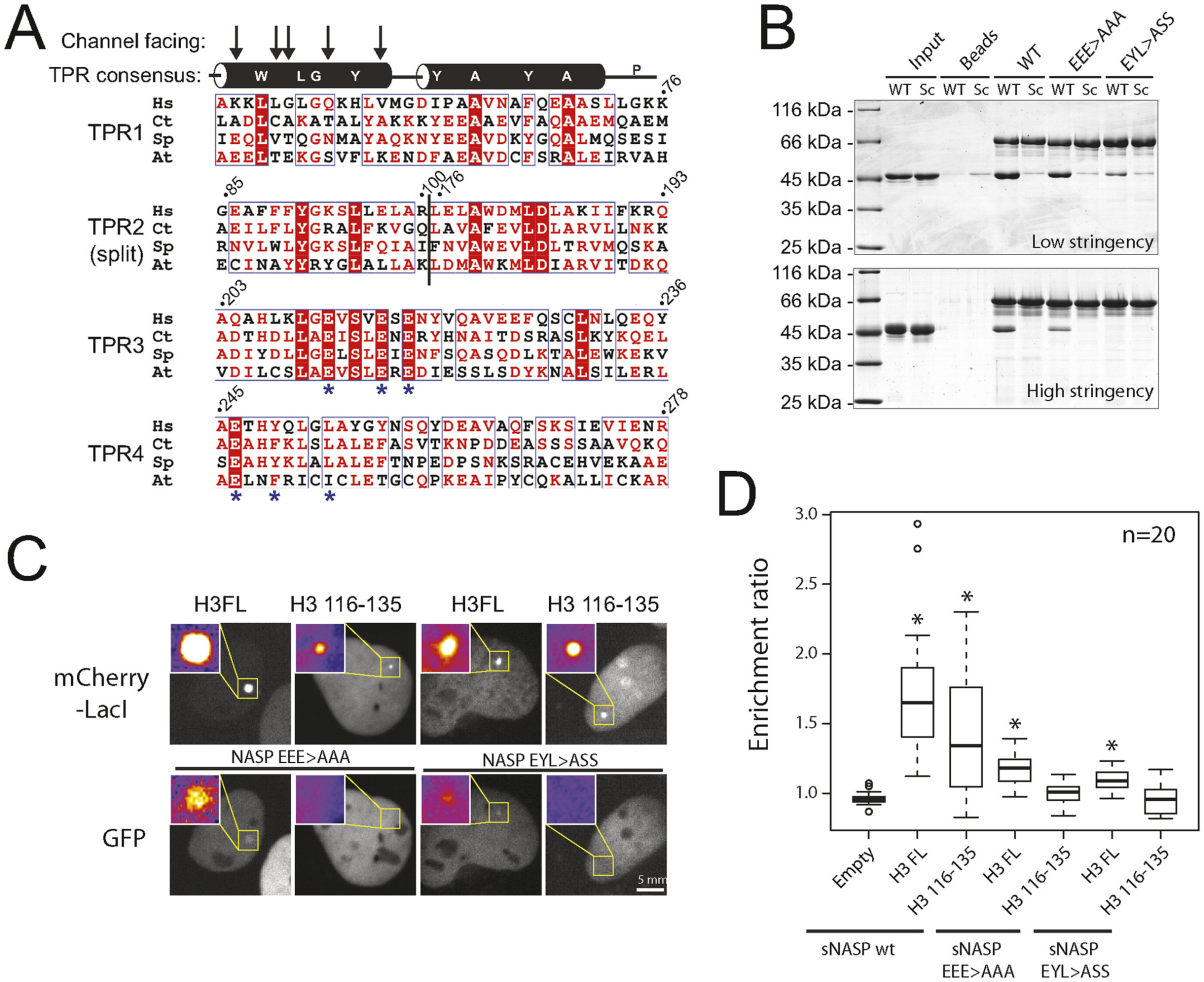
Mutations within the TPR domain of sNASP abolish H3 peptide binding. (**A**) Alignment of the TPR motifs from NASP homologues that retained the H3 116–135 interaction, displayed alongside secondary structural elements and the TPR repeat motif consensus. The positions predicted to face the concave binding channel of the TPR domain fold are indicated by arrows. Asterisks indicate two conserved clusters of residues that are outside of the TPR consensus. Residue numbers for the human homologue are given. The junction of the reconstituted TPR2 is shown as a vertical black line. (**B**) Pulldown experiments of two triple mutants corresponding to the conserved residues identified in (A) under conditions of low stringency (in 20 mM Hepes-KOH pH 7.5, 200 mM sodium chloride) and high stringency (50 mM potassium hydrogen phosphate pH 7.5, 300 mM sodium chloride, 0.1% NP40 and 20% imidazole). (**C**) F2H analysis of GFP-sNASP mutants E211A, E215A, E217A (EEE > AAA) and E246A, Y249S, L253S (EYL > ASS) with mCherry-LacI-H3 or H3 116–135 as indicated. (**D**) Quantification of the enrichment ratio for F2H pairs shown in (C). As comparisons, values for GFP-sNASP recruitment to mCherry-LacI, mCherry-LacI-H3 and mCherry-LacI-H3 116–135 from Figure 1C are shown alongside. Boxes represent the lower quartile, median and upper quartile. Whiskers represent 1.5× the interquartile range. Outliers are indicated by circles. Asterisks indicate a significant difference in enrichment compared to the empty vector control (*P*-value < 0.0125, Wilcoxon rank sum test).

To further substantiate that a canonical TPR-peptide interaction forms when sNASP and H3 interact, and to validate our NMR data, we carried out structure-guided mutagenesis to isolate point mutants within the TPR domain that disrupt binding to the H3 peptide. To aid our chemical shift perturbation mapping of the H3 binding site we also carried out multiple sequence alignment based on our functional analysis of sNASP homologues (Figure 4). The alignment revealed two clusters of conserved residues that colocalized to the region of high chemical shift perturbations (Figure 6A). Based on this analysis, we engineered two triple mutants, E211A/E215A/E217A and E246A/Y249S/L253S of (His)_6_-tagged sNASP to test the importance of these residues in mediating the interaction with H3 116–135. Both mutants pulled down MBP H3 116–135 to a lesser extent than wild type sNASP (Figure 6B), with E246A/Y249S/L253S having a more profound effect than E211A/E215A/E217A. These mutational analyses are supported by our structural findings (Figure 5). All three residues of the EYL>AAA mutant variant show large CSPs and are located directly on the interaction surface, in agreement with these being essential for the sNASP–H3 interaction. Conversely, only two of the three mutated residues of the EEE > AAA variant display significant CSPs; these residues are located on the periphery of the interaction surface and contribute less to the sNASP–H3 peptide binding.

We further tested the interaction of these two sNASP triple mutant constructs using the F2H assay. Mutant sNASP constructs were coexpressed as mEGFP fusion proteins with either mCherry-LacI-H3 or mCherry-LacI-H3 116–135, and their ability to recruit to an integrated LacO array assessed by live cell imaging. As both constructs were fused with mEGFP at their N-termini, and the NLS of sNASP is within its C-terminal region, nuclear localization provides a control that both mutants formed stable fusion proteins. Interestingly, interaction with the H3 116–135 peptide was abolished in both sNASP mutant constructs, whilst interaction with full length H3 was diminished, but remained significantly above background (Figure 6C and D). Whilst these findings confirm that the TPR domain binds the H3 peptide, residual binding of the triple mutant sNASP constructs to full length H3 implies that the sNASP-H3 C-terminal peptide interaction is likely not the only binding site between sNASP and it histone substrate. This idea is consistent with the multivalent protein–protein interactions often seen between histone chaperones and their substrates.

## DISCUSSION

Assembly and deposition of histones into nucleosomes represents a major challenge in ensuring genomic integrity and in regulating many genomic processes. Both an excess of aggregation prone histones, and a lack of packaging substrate represent potentially fatal scenarios. The cell has overcome these potential dangers through the evolution of a highly tuned histone deposition pathway that keeps total soluble histone pools to a minimum whilst providing a constant source of packaging substrate to sites of histone turnover and active replication, even in the face of replicative stress (10,30,57). Many of the underlying mechanics of this histone chaperoning pathway, however, have remained elusive. Although it is clear that numerous proteins are required for a functional nucleosome assembly line, the molecular steps that take histones from their synthesis in the cytosol to the deposition in the nucleus are still largely unknown.

Here we have presented multiple lines of evidence that the TPR motif containing family of histone chaperones, represented by the human sNASP protein, make a high affinity interaction with a discrete peptide epitope located within the globular domain of histone H3. Similar to other chaperone–histone interactions characterized thus far, the residues of H3 that mediate interaction with sNASP are sequestered within the nucleosome structure (Supplementary Figure S4D), offering an explanation as to why sNASP is found predominantly associated with soluble histones. What is surprising, however, is that residues of H3 crucial for sNASP binding are also necessary for the binding of other chaperones, most notably, the highly conserved histone chaperone ASF1 (Supplementary Figure S4D). Structural analysis revealed that ASF1 interacts extensively with the C-terminal region of H3, the same region that we identify as binding to the TPR domain of sNASP (17,58–59. Indeed, a number of residues in H3 that we identify as being crucial for sNASP binding, namely L126 and I130, make up the hydrophobic interface between ASF1 and the H3-H4 dimer. One would assume from this that sNASP and ASF1 are mutually exclusive in their histone binding. However, numerous studies have isolated cellular sNASP and ASF1 in the same histone–bound complex (3,7,9,32).

As a means to reconcile our findings with previous observations (31–32,41,60), we do observe that point mutants within the TPR domain of sNASP, which disrupt binding to an H3 peptide, still retain residual binding to full length H3 when assayed by fluorescence-2-hybrid (Figure 5C). This suggests that sNASP's interaction with its histone substrate is not solely mediated by the peptide binding ability of the TPR domain, but is more complex. Indeed, previous analysis of sNASP's interaction with histones using a number of *in vitro* techniques have identified regions outside of the H3 C-terminal peptide that could mediate secondary interactions with histones (31–32,41,60). We did not detect a direct interaction between sNASP and histone H4, either by F2H analysis or by far western blotting. It is difficult to assess whether H4 tethered to the LacO array folds with endogenous H3, therefore the possibility that sNASP interacts with H4 in the context of an H3-H4 dimer still exists. Further biochemical and structural analysis will be necessary to determine if this is the case.

Another interesting observation that we make here is the ability of sNASP to interact broadly with C-terminal peptides of both canonical H3 and centromeric H3 variants (Figure 3). Previous analysis of the sNASP homologue from fission yeast, Sim3, demonstrated that it could bind both centromeric and canonical isoforms of H3 (38), being identified genetically through mutations in its TPR domain. However, a proteomic approach to identify factors interacting with the human centromeric variant, CENPA, did not find sNASP to be stably associated (16). Whether human sNASP has thus far escaped isolation with CENPA due to only a transient interaction, or whether fission yeast Sim3 is unique in its ability to interact with both canonical and centromeric H3 isoforms, has yet to be determined. Our data indicates that human sNASP may interact with CENPA in specific cellular contexts, such as for example during CENPA incorporation.

It is also worth noting that we could not detect interaction between the budding yeast homologue, Hif1 and the H3 C-terminal peptide *in vitro* (Figure 4), even though we could detect binding for a more distant homologue from plants. Hif1 is well characterized, and has also been shown to adopt other unique features, such as a direct interaction with the co-chaperoning and histone modifying Hat1–Hat2 complex (11,61), an interaction that, to our knowledge, has not yet been reported for any other NASP homologue. Whether such features are functionally linked has yet to be addressed. It does appear, however, that the TPR motif containing family of histone chaperones represented by NASP have diverged much more than other families of chaperones, such as ASF1, and members of the HAT1, CAF1 and HIRA complexes. Interestingly, TPR repeats are also found in the protein TONSL, which promotes homologous recombination and protects cells from replication stress. It would be interesting to test whether the TONSL TPR repeats are involved in histone recognition (62–64).

In summary, our findings identify a novel, TPR repeat-mediated engagement of a histone chaperone with both canonical and non-canonical histone H3 proteins and provides a molecular basis to understand the role of TPR motif containing proteins, such as the histone chaperone sNASP, in chromatin assembly and regulation.

## Supplementary Material

SUPPLEMENTARY DATA
