# Supplementary material for: The histone chaperone sNASP binds a conserved peptide motif within the globular core of histone H3 through its TPR repeats
Source: Nucleic Acids Res. 2015 Dec 15;44(7):3105–17. doi: 10.1093/nar/gkv1372 (PMC4838342; doi:10.1093/nar/gkv1372)
Supplement: SUPPLEMENTARY DATA [file supp_44_7_3105__index.html]

The histone chaperone sNASP binds a conserved peptide motif within the globular core of histone H3 through its TPR repeats — The histone chaperone sNASP binds a conserved peptide motif within the globular core of histone H3 through its TPR repeats — SUPPLEMENTARY DATA 

# The histone chaperone sNASP binds a conserved peptide motif within the globular core of histone H3 through its TPR repeats

## SUPPLEMENTARY DATA

- SUPPLEMENTARY DATA
