# Supplementary material for: The histone chaperone sNASP binds a conserved peptide motif within the globular core of histone H3 through its TPR repeats
Source: Nucleic Acids Res. 2015 Dec 15;44(7):3105–17. doi: 10.1093/nar/gkv1372 (PMC4838342; doi:10.1093/nar/gkv1372)
Supplement: SUPPLEMENTARY DATA [file supp_gkv1372_nar-02972-m-2015-File008.pdf]

Supplementary Figure 1

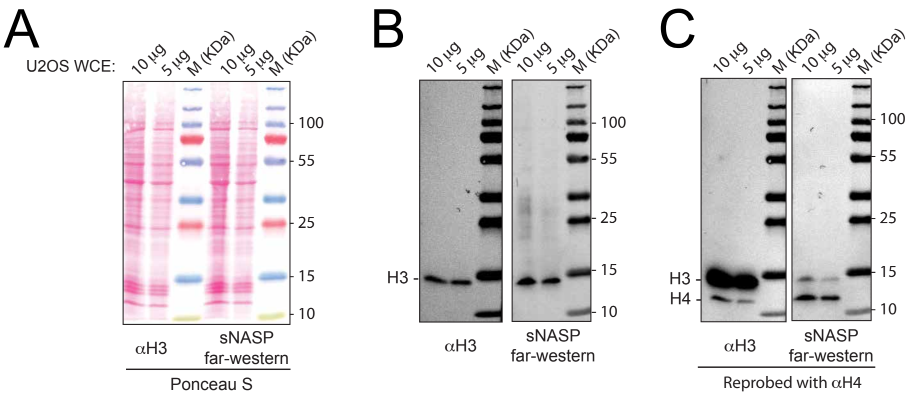

Supplementary Figure 1. Far-western blotting of whole cell extracts (WCE) prepared from the U2OS cell line. (A) WCE prepared from U2OS cells stained with ponceau. The abundant core histones are visible as four discrete bands resolved between the 10 and 15 kDa markers. (B) The same blot was either probed with biotinylated sNASP or an anti-H3 antibody (Abcam: ab1791). Immobilised polypeptides which bound to sNASP were then visualised by streptavidin-HRP. sNASP recognises a band that migrates at the same position as H3 dected by and H3 antibody. (C) To show that the band recognised as H3 was distinct from H4, the blots were reprobed with an H4 antibody, which detects a faster migrating species.

Supplementary Figure 2

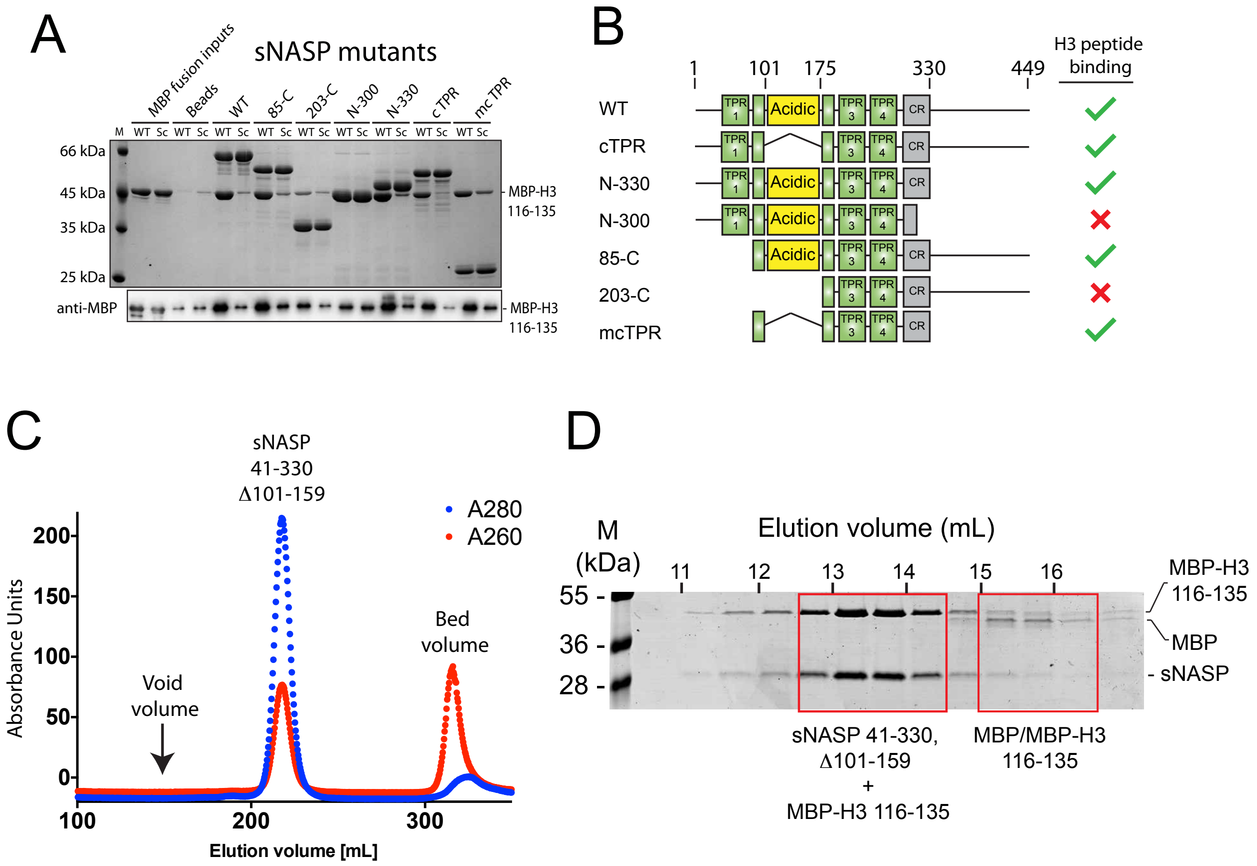

Supplementary Figure 2. Truncation analysis of sNASP & biochemical validation of the sNASP 41-330, Δ101-159 construct used for NMR (A) Various truncations of (His)6-sNASP were tested for their ability to pull down MBP -H3 116-135 (WT) or a scrambled peptide (Sc). After washing, bound sNASP truncations and MBP-peptide fusions were separated by SDS-PAGE and stained with Coomassie. As sNASP N-300 and MBP-peptide fusions ran at the same position, western blotting for MBP was also carried out. (B) Domain diagrams of truncations shown in A with TPR motifs in green, the acidic loop region in yellow and the predicted capping region in grey. A summary of the interaction with MBP-H3 116-135 is shown on the right hand side. (C) Gel filtration analysis of ^{15}N labelled sNASP 41-330, Δ101-159 showed a stabile monodisperse elution profile. (D) Co-elution of sNASP 41-330, Δ101-159 with MBP-H3 116-135 during gel filtration chromatography, demonstrating the sNASP truncation retains its H3 peptide binding ability. MBP, Maltose Binding Protein; CR, capping region; TPR, tetratricopeptide repeat; cTPR, contiguous TPR; mcTPR, minimum contiguous TPR.

Supplementary Figure 3

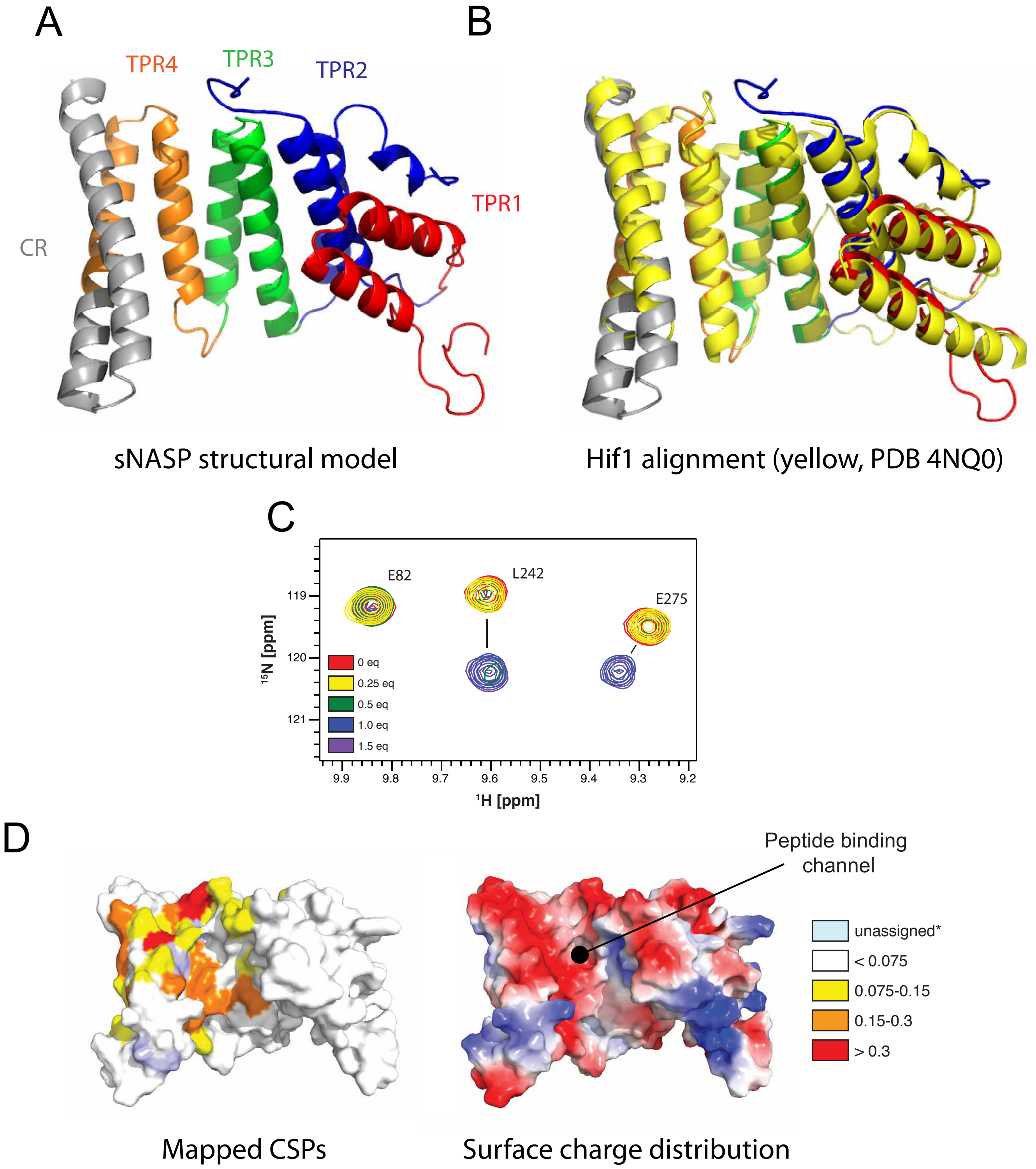

Supplementary Figure 3. A homology model of sNASP based on Hif1p. (A) The structural model of sNASP generated using http://swissmodel.expasy.org/ with the budding yeast homolog Hif1p as a scaffold and NMR data. The TPR domain and capping region (CR) are coloured differently to aid in visualisation. Residues that were shown to disrupt H3 peptide binding are shown in cyan. (B) Alignment of Hif1p (yellow) to the homolgy model of sNASP. (C) Overlay of a zoomed in region from the ^{1}H-^{15}N HSQC spectra of the apo and H3 peptide (115-135) bound sNASP (41-330, Δ101-159), coloured from red to blue, respectively. Residues L242 and E275 lie within the TPR3-4 region and show significant CSP, whereas E82 lies within the TPR1-2 region and does not. (D) The surface charge distribution of the sNASP homology model calculated using the protein contact potential function in PyMol. Regions of negative charge are shown in red, whereas regions of positive charge are shown in blue. The H3 peptide binding channel is annotated.

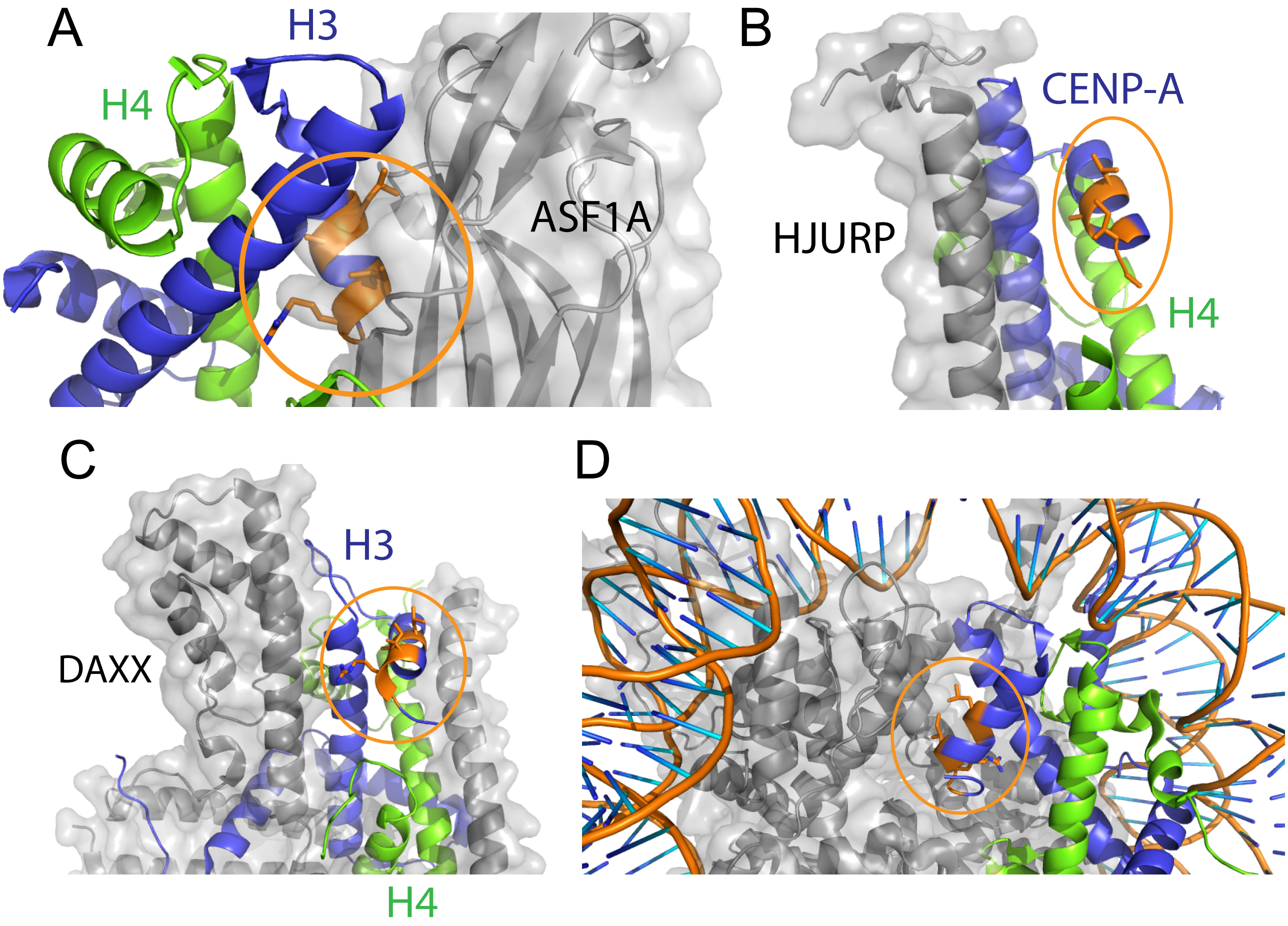

Supplementary Figure 4. A comparison of sNASP's interaction site with other known structures containing histones H3 and H4. The five residues that are critical for the binding of sNASP to the H3/CENPA C-terminal region are shown mapped onto structures of H3/CENPA-H4 complex with other histone chaperones, or the nucleosome. (A) ASF1 (PDB code 2HUE), (B) HJURP (PDB code 2YFV), (C) DAXX (PDB code 4H9N), and (D) the nucleosome (PDB code 1KX5). H3 is shown in blue, H4 in green, whilst the chaperones and nucleosome are shown in gray. In the nucleosome structure only a single H3-H4 dimer is shown for clarity. Residue of the H3 epitope bound by sNASP are shown in orange and are circled.
